# Bone Marrow GvHD after Allogeneic Hematopoietic Stem Cell Transplantation

**DOI:** 10.3389/fimmu.2016.00118

**Published:** 2016-03-30

**Authors:** Martin Szyska, Il-Kang Na

**Affiliations:** ^1^Experimental and Clinical Research Center (ECRC), Berlin, Germany; ^2^Department of Hematology, Oncology and Tumor Immunology, Charité – Universitätsmedizin Berlin, Berlin, Germany

**Keywords:** GvHD, bone marrow, alloHSCT, memory niche, minor antigen, GvL, GvT, bone marrow stroma

## Abstract

The bone marrow is the origin of all hematopoietic lineages and an important homing site for memory cells of the adaptive immune system. It has recently emerged as a graft-versus-host disease (GvHD) target organ after allogeneic stem cell transplantation (alloHSCT), marked by depletion of both hematopoietic progenitors and niche-forming cells. Serious effects on the restoration of hematopoietic function and immunological memory are common, especially in patients after myeloablative conditioning therapy. Cytopenia and durable immunodeficiency caused by the depletion of hematopoietic progenitors and destruction of bone marrow niches negatively influence the outcome of alloHSCT. The complex balance between immunosuppressive and cell-depleting treatments, GvHD and immune reconstitution, as well as the desirable graft-versus-tumor (GvT) effect remains a great challenge for clinicians.

For many decades, allogeneic stem cell transplantation (alloHSCT) has been used for the treatment of hematological malignancies. Alloreactive T cells contained within the donor bone marrow preparations have eventually been recognized as both causative for the life-threatening graft-versus-host disease (GvHD) and the beneficial graft-versus-tumor (GvT) effect (1, 2). Great scientific effort has since been put into further delineating the impact of T cell subpopulations and associated effector functions on GvHD development in order to segregate GvHD from GvT but no clinically feasible solution to this apparent dilemma has yet emerged.

Skin, liver, and intestine are regarded as the principal target organs of GvHD that can be affected to varying degrees or not at all. Individual outcomes of alloHSCT are hardly predictable because the complex interplay of multiple factors is just starting to be understood. GvHD is commonly correlated with long-term cytopenic conditions, resulting in mortality due to infections and bleeding complications (3). Besides toxicity resulting from conditioning treatment, alloreactivity in the bone marrow has been deemed responsible for the observed defects in hematopoiesis. Recent studies conclusively demonstrated niche-forming cells in the bone marrow as targets of GvHD (4, 5).

Here, we address the molecular and cellular causes of GvHD in general and focus next on the sequence of events leading to hematopoietic failure and immunodeficiency as a consequence of alloreactivity in the bone.

## Source of Alloreactivity

In both GvHD and GvT, donor T cells react against host cells expressing alloantigens. In major histocompatibility complex (MHC)-mismatched alloHSCT, a large fraction of donor T cells targets monomorphic host proteins presented as peptides in the context of recipient MHC molecules. In donors, developing T cells are negatively selected exclusively against proteins presented as peptides bound to self-MHC molecules. Therefore, a large fraction of these T cells express T cell receptors with high affinity for host MHC molecules or the presented peptides in their context (6). Due to the comparably large number of reactive T cell clones (7), the ensuing GvHD response in MHC-mismatched settings is usually very severe and can be difficult to control despite the application of intensive immunosuppressive treatments.

In MHC-matched transplant settings, donor T cells target minor histocompatibility antigens (MiHAs), polymorphic genes presented *via* MHC molecules as processed peptides. Negative selection against these antigens is absent in the donor thymus due to lack of expression. Therefore, T cell receptors with high affinity to recipient MiHAs exist in small frequencies within the donor T cell repertoire. Respective T cell clones can become activated in an inflammatory environment as caused by pretransplant regimens and may trigger GvHD or react against MiHA-expressing tumor tissue. Whereas in MHC-mismatched alloHSCT, alloantigens are exclusively presented by host antigen-presenting cells (APCs), in an MHC-matched setting they can additionally be of donor origin due to cross presentation after uptake of host cell fragments (8, 9). Although only a fraction of polymorphic genes can be presented as peptide in a given MHC combination and single MiHA differences are not regarded as sufficient for the induction of GvHD in clinical settings, novel tools such as global genome association studies and *in silico* prediction have been widely used to identify an ever-growing set of clinically relevant MiHAs among thousands of polymorphic genes (10, 11), explaining the high incidence of GvHD even in MHC-matched transplantations.

Alloreactive donor T cells exert their effector function *via* both soluble and cell-contact-dependent cytotoxic factors. Upon activation by APCs, mainly CD4 T cells produce Th1-type cytokines, including interferon-γ (IFN-γ), tumor-necrosis factor-α (TNF-α), and interleukin-1 (IL-1). These soluble factors are systemically transported through the blood to GvHD target organs and locally act by rendering various cell types more susceptible to the ensuing alloreactive T cell response.

Antigen-specific target cell killing is principally mediated by the perforin–granzyme pathway and Fas–Fas ligand (FasL) interaction, both of which are employed by both CD4 and CD8 cytotoxic T lymphocytes (CTLs). Upon binding to their cognate antigen, CTLs can secrete perforin and granzyme, which in combination leads to lysis and rapid apoptosis of target cells. In an inflammatory context, Fas can be upregulated on target cells making them susceptible for cytotoxic killing by FasL-expressing T cells (12).

Due to broad functional overlap, complex differential expression of Fas in various organs under different pretreatment conditioning and incompatible GvHD models used in respective studies, the individual impact of FasL and perforin–granzyme pathways from CD4 and CD8 effectors is still under discussion (13). However, the FasL–Fas pathway appears to be more associated with the establishment of donor chimerism and GvHD severity and more important for CD4 effector function, whereas GvT is suggested to be more attributable to perforin–granzyme cytotoxicity without any salient T cell subset preference (14–16).

Ultimately, even a complete lack of both cytolytic pathways does not abolish GvHD, clearly demonstrating the existence of additional cytotoxic effectors in the T cell arsenal (17). TNF-related apoptosis-inducing ligand (TRAIL) and TNF-like weak inducer of apoptosis (TWEAK) are both expressed by T cells and have been assumed to partially compensate under these conditions by signaling through their cognate receptors death receptor 4 (DR4) or DR5 and TWEAK receptor (CD266), respectively (18).

## GvHD and Immune Reconstitution

In order to facilitate bone marrow engraftment and to diminish tumor burden, patients are treated with conditioning prior to alloHSCT, leading to tissue damage and immunosuppression. Whereas acute GvHD (aGvHD) occurs in the early phase after alloHSCT or immediately after termination of immunosuppressive regimen, chronic GvHD (cGvHD) emerges later, is clinically less defined and shares many characteristics with autoimmune diseases, including *de novo* generation of autoreactive T cell clones and the development of autoantibody titers (19, 20).

In affected patients, harsh treatment with immunosuppressive drugs can control the effects of GvHD, though at the cost of delayed immune reconstitution and mitigation of desired GvT effects. Even without immunosuppression, outcomes of sublethal GvHD include generalized cytopenia and a dramatically delayed immune reconstitution of all lymphoid lineages (21). Consequently, lethal GvHD and opportunistic infections are responsible for high mortality in relapse-free patients within the first year after alloHSCT (22, 23).

The discovery of strong alloreactivity against thymic tissues has suggested a link between aGvHD and impaired T cell reconstitution after alloHSCT (24). Allogeneic T cells targeting host T cells and also the thymic architecture can easily be understood as causative for T cell deficiencies with additional implications for B cell immunity, since both B cell effector function and memory formation largely depend on interaction with CD4 T cells (25). Thymic GvHD also adds a new layer of understanding on the frequently observed autoimmune traits of ensuing cGvHD. The thymus is the principal organ of T lymphocyte development, in charge of generating an extremely diverse set of T cell clones, while eliminating autoreactive clones (26). Arguably, the stringent and finely tuned T cell selection process in the thymus can be unhinged by destruction of self-APCs, which could allow potentially autoreactive clones to escape into the periphery and to cause autoimmune symptoms.

Using more refined mouse models of both MHC-matched and mismatched HSCT, the bone marrow has been established as an additional target of GvHD. In these studies, GvHD generally affected hematopoiesis and lymphoid development (27). However, serial bone marrow transfer experiments strongly suggest that GvHD progression depends on the targeting of non-hematopoietic cells of the bone marrow (5). Infiltrating T cells of donor origin were clearly associated with both impaired hematopoiesis and destruction of specialized niche-forming cells, including osteoblasts and sinusoidal vascular endothelial cells (28). In several studies, GvHD effects seemed to selectively impair B cell development (14, 29). For the first time, Mensen et al. could translate these findings to the human setting by correlating impaired immune reconstitution after HSCT with both GvHD and T cell infiltration into the bone marrow and by demonstrating a striking reduction in osteoblasts in these patients (4). This closely resembles the findings in mouse models of GvHD allowing a generalized view on immunodeficiency after alloHSCT.

## Implications of Bone Marrow Niche Destruction

In adult individuals, the bone marrow is both the origin of hematopoiesis and the ultimate harbor of immune cells comprising the immunological memory, namely, long-lived plasma cells and memory CD4 and CD8 T cells (30–34). Furthermore, hematological malignancies either originate or later become manifest in the bone marrow. All immunological functions strictly depend on a complex organization of niche-forming stromal cells of mesenchymal and endothelial origin providing important developmental cues to hematopoietic progenitors or crucial survival signals to memory cells.

**Figure 1:**
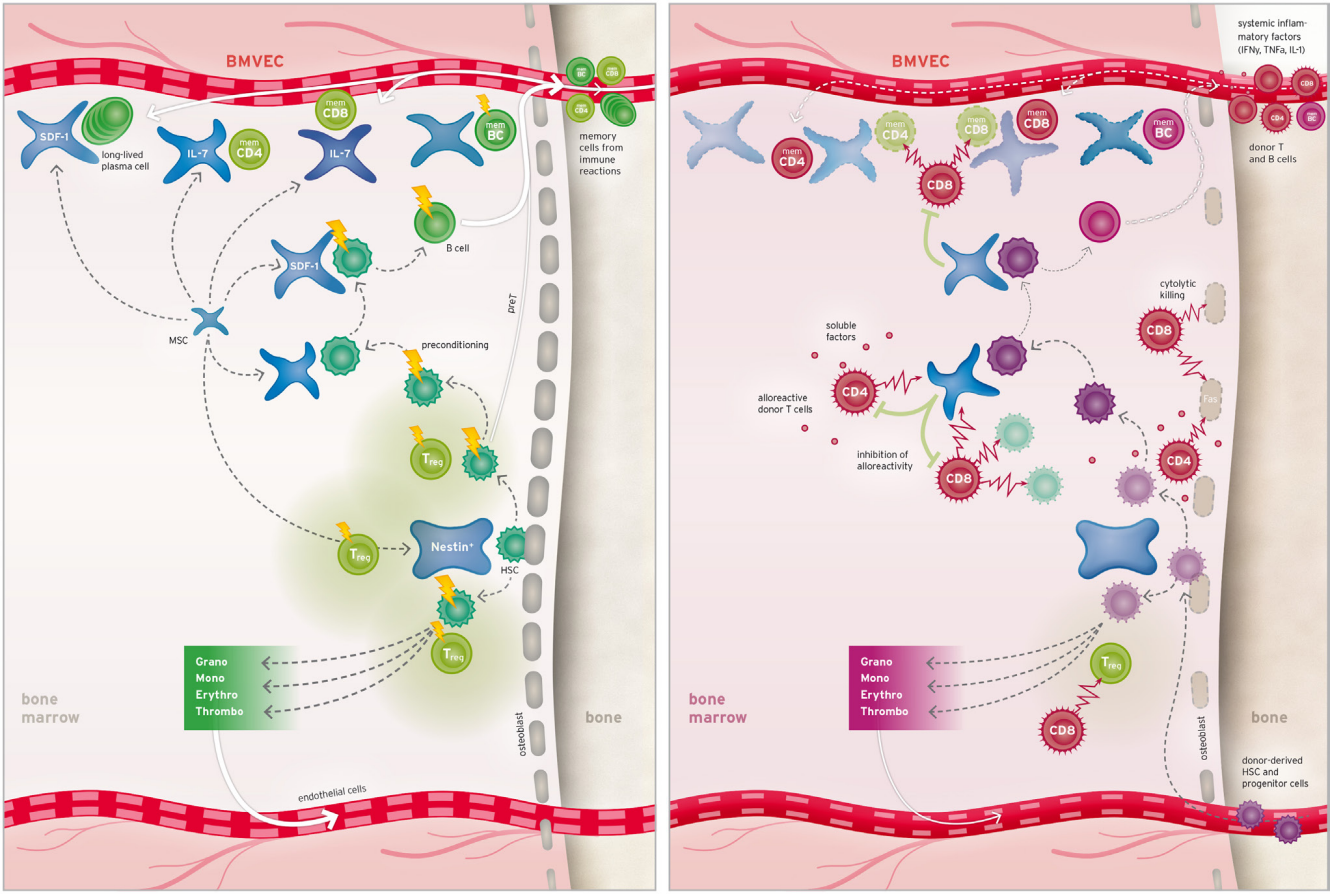
**Bone marrow niches and their response to preconditioning and alloHSCT**. Left: niches for hematopoietic stem cells (HSCs) are located at the endosteal border and comprise osteoblasts and specialized mesenchymal cells partly immunoprotected by adjacent regulatory T cells (Treg). Alternatively, HSCs can seed into perivascular niches (not shown). B cell progenitors and recirculating memory T, B (memCD4, memCD8, and memBC), and plasma cells occupy additional specialized perivascular niches. Upon preconditioning, bone marrow cells are differently affected by the treatment as indicated by the flash size. Right: after alloHSCT, alloreactive T cells are massively activated (not shown) leads to systemic influx of soluble inflammatory factors into the bone marrow where they cause upregulation of Fas on various cell types and harm donor and recipient HSCs alike. Infiltrating alloreactive donor T cells deplete residual host hematopoietic cells and support disintegration of endostial and perivascular niches by means of cytolytic and soluble factors. Consequently, efflux of hematopoietic lineages and seeding capacity for donor-derived hematopoietic stem and memory cells is diminished.

Figure 1 proposes a model of bone marrow GvHD by bringing together data from clinical studies and various scientific investigations using mouse models or *in vitro* culture systems. Figure 1 (left) depicts bone marrow homeostasis with a focus on niche constituents and points out cells susceptible to standard preconditioning treatment. Figure 1 (right) shows cell types and effector mechanisms involved in acute bone marrow GvHD and clarifies how multiple niches are impacted by alloreactivity as explained in the following section.

A mutual starting point for all hematopoietic lineages is the endosteum, where self-renewing hematopoietic stem cells (HSCs) reside in niches made up of specialized osteoblasts and nestin-expressing MSCs (35, 36) presumed to be osteogenic progenitors. Alternatively, HSCs can also be maintained in perivascular niches made up of CXCL12-expressing MSCs adjacent to vascular endothelial cells of bone marrow sinusoids (37, 38). Although both the endosteal and the perivascular niche seem to equally support HSC maintenance, the interplay between both niches has not been established yet (39). During proliferation of niche-derived cells, individual lineage decisions are made in a step-wise fashion during migration from the endosteum toward the marrow sinusoids, where cells ultimately exit the bone marrow and enter the blood circulation. For B cells, the maturation from HSC to transitional B cell exiting the bone marrow requires multiple dedicated stromal cells providing stage-specific signals (40).

Stromal and hematopoietic cells are differently affected by the conditioning treatment prior to alloHSCT. HSCs, hematopoietic progenitor cells, B cells, myeloid cells, and, to a lesser extent, T cells are depleted by irradiation and/or anti-mitotic drugs (27).

In contrast, mesenchymal and endothelial cells as well as memory T and B cells are resting cells and were shown to be comparably resistant to depletion (41, 42). Danger signals mediated by tissue damage trigger APC maturation and increased presentation of alloantigens *via* MHCI and II.

Upon alloHSCT, donor T cells, contained within the bone marrow preparations, circulate into secondary lymphoid organs and become activated mainly *via* interaction with dendritic cells (DC), APCs that express alloantigens along with high levels of the costimulatory molecules CD80 and CD86 upon preconditioning (9). At least for CD8 T cells, the initiation of aGvHD is strictly dependent on host APC activity (43). Activated T cells start proliferating and secrete large amounts of inflammatory cytokines and thereby initiate the acute phase of GvHD.

In the bone marrow, systemic inflammation leads to drastic changes in bone marrow-resident cells. TNF-α and IL-1 signaling stimulates upregulation of MHCII, CD40, and adhesion signals in endothelial cells, and blood vessel walls become more permeable, facilitating activation and entrance of innate immune cells and alloreactive lymphocytes into the bone marrow (44). Furthermore, prolonged systemic levels of IFN-γ in combination with TNF-α initiate endothelial cell death, which can be expected to impair maintenance of perivascular niches for memory and stem cells (45).

Osteoblasts constitutively express MHCI and potentially respond to inflammatory signals by upregulation of MHCII, Fas, and CD40, making them easy targets for alloreactive T cell response (46, 47). Mesenchymal cells also respond to IFN-γ by upregulation of MHCI and MHCII but demonstrate a striking inability to activate alloreactive T cells (48, 49). Even more, mesenchymal cells are capable of actively inhibiting T cell effector function by direct and indirect mechanisms, including interference with DC maturation and secretion of IL-10 (50). However, it is unclear whether MSCs themselves have the potential to resist strong alloreactivity. In a likely scenario, perivascular MSCs constituting important survival niches for memory and HSCs would at least display reduced niche capacities when adjacent endothelial cells became apoptotic.

Concomitantly, donor HSCs and progenitor cells enter the bone marrow *via* sinusoids and migrate to cell-specific niches that have been made available by depletion of host hematopoietic cells. However, HSC survival and proliferative capacity is also affected by soluble inflammatory factors, resulting in reduced HSC seeding under aGvHD conditions (51, 52).

Subsequently, activated alloreactive donor T cells infiltrate the bone marrow and exert their cytolytic effector functions by attacking cells presenting host alloantigens. The outcome of the ensuing immune response is impacted by multiple factors, including the immunogenic strength, level of presentation and number of individual alloantigens on host cells, the degree of inflammation caused by conditioning treatment, the size of the residual host T cell population capable of mounting a host-versus-graft response, and the naive repertoire of donor T cells.

Ultimately, the remaining hematopoietic host cells, including HSCs and memory cells, are depleted mainly *via* CD8-derived Fas–FasL and complete donor chimerism is established (14, 27). At that stage, the patient’s immunological memory should be deleted with grave implications for immunity against recurring pathogens. As recipient APCs of hematopoietic origin are depleted, alloantigen is largely presented by donor-derived APCs. However, due to the poor engraftment potential of donor-derived mesenchymal cells, the majority of niche-forming cells in the bone marrow remain host mesenchymal and endothelial cells continuously expressing alloantigens (53). Osteoblasts and endothelial cells present MHCI and II at least under inflammatory conditions and therefore, most niche-forming cells of the bone marrow, albeit poor antigen presenters, constitute targets of alloreactivity under harsh conditions such as preconditioning and aGvHD (47). Additionally, continuous presentation of phagocytosed alloantigens by donor-derived APCs *via* MHCII, and to a lesser extent *via* cross presentation to MHCI, can support indirect niche destruction mainly *via* alloreactive CD4 T cells secreting soluble factors.

In sum, bone marrow GvHD leads to gradual reduction of aforementioned niches, which diminishes hematopoiesis and seeding of donor-derived memory cells into their respective bone marrow niches (Figure 1, right). Although the endosteal niche has recently been reported to be immune-privileged by means of regulatory T cells (54), steep reduction in osteoblast numbers argues against any protective environment at least under GvHD conditions. The bone marrow niche size for distinct cell populations is strictly limited by the number of respective niche-forming cells; and hence, any reduction in their numbers directly decreases the specific niche-capacity of the bone marrow. IL-7-expressing perivascular stroma cells in the bone marrow comprise the niche for memory T cells and loss of these cells due to alloreactivity negatively impacts donor memory T cell seeding and prolongs the preexisting immunosuppressive state of patients receiving alloHSCT (31, 55). Furthermore, it is not surprising that the B cell developmental program, requiring several distinct populations of niche-forming cells for individual maturation steps, proves to be the most affected cell population (29, 40). Hypothetically, the tightly regulated process of central tolerance that removes potentially autoreactive B cell clones could readily be unhinged by GvHD-mediated niche damage, possibly leading to secondary autoimmune symptoms synonymous with the chronic form of GvHD. Interestingly, cGvHD has been shown to be associated with autoantibody titers (56). All factors described above work in combination at manifesting a stage of prolonged immunosuppression.

## Future Directions

The rationale of using alloHSCT for the treatment of hematological disorders is the rapid replacement of the patient’s defective hematopoietic system, whereas harnessing the alloreactivity of donor T cells for rejection of persistent tumor. However, these very T cells are also responsible for GvHD and accelerated immunodeficiency, a price we are willing to pay for a chance of relapse-free survival. However, the hidden cost is obviously much higher, considering that donor T cells are also responsible for bone marrow GvHD, which potentially leads to extensive destruction of niche-forming cells by not yet fully understood mechanisms. This greatly influences the kinetics of comprehensive immune reconstitution, because the replenishment of destroyed niches is apparently very slow. Besides broad effects on hematopoiesis, the seeding capacity of recirculating memory T cells into the bone marrow might be impacted by bone marrow GvHD, as we could show for B cell development. In consequence, a large fraction of treated patients remains vulnerable to otherwise harmless infections for months or years after treatment. Further research is needed to better protect dedicated bone marrow niches from GvHD. Alternatively, novel transplantation protocols should demonstrate improved seeding capacity of donor mesenchymal stem cells to rapidly replenish destroyed host niche-forming cells and to harness their unique immunosuppressive properties (57).

## Author Contributions

MS wrote the mini review. IN supervised, edited and approved the final version for publication.

## Conflict of Interest Statement

The authors declare that the research was conducted in the absence of any commercial or financial relationships that could be construed as a potential conflict of interest.
